# Disinfectants

**Published:** 1874-08

**Authors:** 


HALL'S

### JOURNAL OF HEALTH

AND

MISCELLANY.

VOL. XXI.—AUGUST, 1874.—NO. VIII.

### DISINFECTANTS.

THE only certain, speedy and permanent disinfectant known to man is cleanliness.

Antiseptics are substances which arrest and prevent decay, such as creosote. Deodorizers take away the ill smell attached to localities, arising from decomposition, either by absorption, as pulverized charcoal, or by forming new chemical combinations, as copperas. But many deodorizers have an odor of their own which overpowers that which was sought to be got rid of; among these are burning tar, and brown sugar sprinkled on burning coals.

Disinfectants are substances which take away the power of conveying disease. The emanations from what passes out of the body of persons having cholera cause the disease in others; but if carbolic acid is instantly thrown upon these dejections they are rendered harmless. Common copperas, called sulphate of iron, in its crude state can be purchased for five cents a pound; this dissolved in two gallons of water, and thrown over ill smelling places, is the cheapest, simplest and most convenient deodorizer, and is applicable to privies, sinks, cellars, gutters and heaps of offal. Common fresh dug earth is efficient, plentifully sprinkled over offensive places.

A cheap and easily available disinfectant and deodorizer is made by dissolving a bushel of salt in a barrel of water, then add enough unslacked, that is fresh lime, which has never been exposed to dampness, to make the whole into a thin paste, to be applied as often as necessary to all places yielding offensive smells, such as gutters, sinks, cesspools and the like; this is home made chloride of lime.

But as all disinfectants, deodorizers and antiseptics must be applied from time to time, it is less trouble and a greater wisdom to sedulously cultivate habits of the strictest cleanliness in person, clothing and habitation, indoor and out; as well in the cellar as in the parlor; as well in the darkest closet as in the hall; not neglecting a corner or a crevice in the whole building, keeping an eye to one point always, that wherever there is dampness there is disease, and that moral purity and filth in any form are absolutely incompatible.

